# New Formulations of Local Anaesthetics—Part I

**DOI:** 10.1155/2012/546409

**Published:** 2011-12-05

**Authors:** Edward A. Shipton

**Affiliations:** Department of Anaesthesia, University of Otago, Christchurch 8042, New Zealand

## Abstract

Part 1 comments on the types of local anaesthetics (LAs); it provides a better understanding of the mechanisms of action of LAs, and their pharmacokinetics and toxicity. It reviews the newer LAs such as levobupivacaine, ropivacaine, and articaine, and examines the newer structurally different LAs. The addition of adjuvants such as adrenaline, bicarbonate, clonidine, and corticosteroids is explored. Comment is made on the delivery of topical LAs via bioadhesive plasters and gels and controlled-release local anaesthetic matrices. Encapulation matrices such as liposomes, microemulsions, microspheres and nanospheres, hydrogels and liquid polymers are discussed as well. New innovations pertaining to LA formulations have indeed led to prolonged action and to novel delivery approaches.

## 1. Introduction

Local anaesthetics (LAs) are used clinically for anaesthesia and analgesia either following surgery or for management of other acute and chronic pain conditions; they only last a few hours. Part 1 of this paper deals with the newer LAs, more recent LA formulations, a better understanding of the mechanisms of action of LAs, and their pharmacokinetics and toxicity.

Local anaesthesia for a prolonged period of days is best provided using catheter techniques [1] with disposable pumps [2] or multiple injections [3]. Most attempts to prolong LA action have so far only doubled or tripled the plain drug effect time, using adjuncts to LA agents of readily available agents. These include opioids and clonidine that delay local anaesthetic clearance from their site of action [4] and dexamethasone that prolongs peripheral nerve and plexus blocks [5].

## 2. Types of Local Anaesthetics

Lignocaine is perhaps most commonly used or known local anaesthetic agent; it is used either in local or regional anaesthesia, or in epidural or spinal blockade; it has a number of uses in anaesthesia and pain medicine. However, it is also given parenterally in the management of neuropathic pain states. EMLA, a eutectic mixture of lignocaine and prilocaine, is an effective topical anaesthetic in preventing pain associated with needle procedures [6].

**Figure 1:**
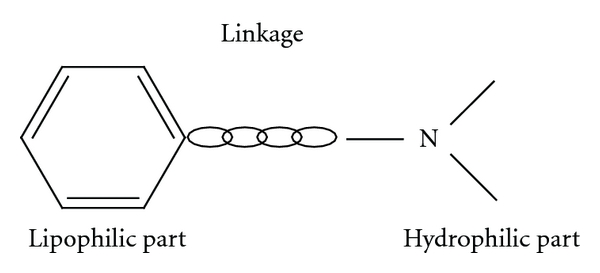
Structure of all local anaesthetics.

**Figure 2:**
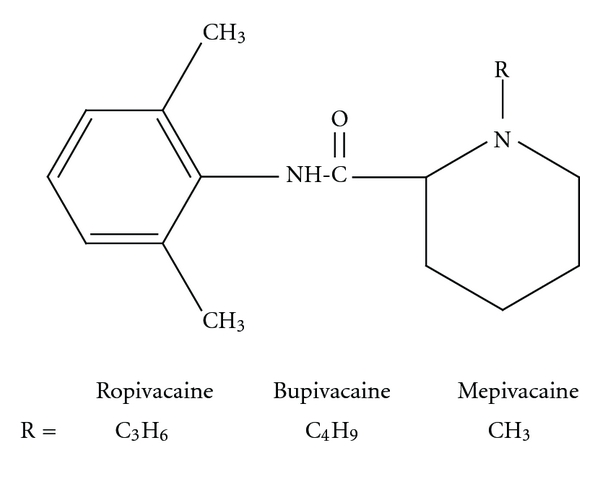
Amide local anaesthetics.

Local anaesthetics can be classified into two groups based on the nature of the link, namely, amides [–NH–CO–] and esters [–O–CO–] (Figures 1 and 2). The amide group is the most commonly used clinically; it includes lignocaine, prilocaine, levobupivacaine, bupivacaine, mepivacaine, and ropivacaine.

**Figure 3:**
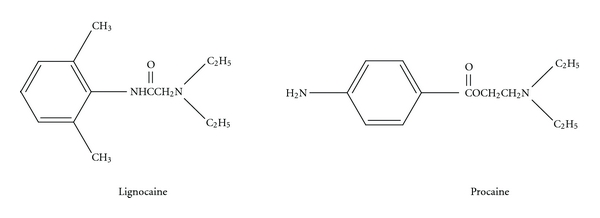
Amide local anaesthetic (lignocaine) and ester local anaesthetic (procaine).

The ester group is weak bases, solubilised for injection as strong conjugate acidic hydrochloride salts (pH 3–6); it includes cocaine, procaine, chloroprocaine, and amethocaine (Figure 3) [3, 7]. Benzocaine and butamben are ester-type local anaesthetics mostly used in topical and mucous formulations. Anaphylaxis to local anaesthetics is very uncommon and has decreased in frequency because of the decreasing use of the ester group of local anaesthetics [8]. Most allergic reactions are due to the common metabolic product of the ester local anaesthetic, para-amino benzoic acid [8]. Cross-reactivity among esters is common. Allergic reactions to amide local anaesthetics remain anecdotal. Ingredients included in local anaesthetic solutions such as antioxidants or preservatives including metabisulphite or parabens (also metabolised to para-amino benzoic acid) may also elicit allergic or adverse reactions [8]. Local anaesthetics (without preservatives or adrenaline) may be skin tested.

## 3. Pharmacokinetics of Local Anaesthetics

Injectable local anaesthetics are subject to absorption; a large fraction of the injected drug is removed by the systemic circulation and distributed to distant organs according to their vascular density [9]. Highly vascular organs (brain, heart, lung, liver, and kidneys) are exposed to unmetabolised local anaesthetic at peak concentration. The local anaesthetic is taken up within each organ according to its tissue-plasma partition coefficient. Most absorbed local anaesthetic is cleared from the liver. Hepatic clearance is a function of the hepatic extraction ratio and hepatic blood flow. The hepatic extraction ratio, in turn, is dependent on the ratio of free to protein-bound drug. Local anaesthetics bind tightly to plasma proteins greatly limiting the free fraction of available drug. Only the free or unbound fraction that is bioactive. Like most weak bases, local anaesthetics bind mainly to alpha-1-acid glycoprotein. Lignocaine, being moderately protein-bound, has a high hepatic extraction ratio (70–75% per pass) [9]. Clearance is therefore flow-limited and is reduced by factors that limit hepatic blood flow. Conversely, bupivacaine and ropivacaine, being highly protein-bound, are cleared <50% per pass; hence, their clearance depends on free drug concentration [9]. Low cardiac output states may not greatly affect the plasma concentration of the highly protein-bound agents, as their clearance is not flow limited. Intrinsic hepatic disease may alter clearance by altering plasma protein content and degree of protein binding, by decreasing the enzyme activity of the liver, and by reducing hepatic blood flow. Patients with liver disease may have single-shot blocks with normal doses. Doses for continuous infusion and repeat blocks need to be significantly reduced (10–50% relative to the degree of dysfunction) due to the risk of accumulation of the primary compound and its metabolites [10]. Patients with mild or controlled cardiac failure may not need a dose reduction for single-shot blocks. Doses of ropivacaine and bupivacaine for continuous infusion and repeat blocks need to be reduced, as their metabolites will be eliminated slowly. In patients with renal dysfunction, reduced clearance and faster absorption of local anaesthetic lead to an elevation in plasma concentration [10]. Clearance of both bupivacaine and ropivacaine has been shown to be reduced in uraemic patients [9, 10]. The clearance of one of the main metabolites of ropivacaine, 2,6-pipecoloxylidide (PPX), is also decreased in uraemic patients [9].

## 4. Newer Local Anaesthetics

### 4.1. Levobupivacaine

**Figure 4:**
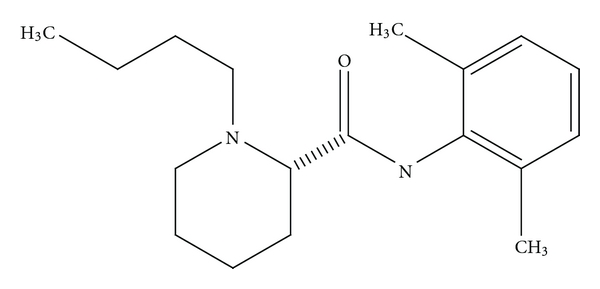
Structure of levobupivacaine.

In recent years, levobupivacaine, the pure S (−) enantiomer of bupivacaine, emerged as a safer alternative for regional anaesthesia than its racemic parent (Figure 4). In common to all local anaesthetics, levobupivacaine reversibly blocks the transmission of action potential in sensory, motor, and sympathetic nervous fibres by inhibiting the passage of sodium through voltage-sensitive ion channels in the neuronal membrane. Various factors such as site of administration, duration of continuous infusion, and/or addition of agents with vasomotor effect may influence the degree of systemic uptake of levobupivacaine.

In pharmacodynamic studies, levobupivacaine demonstrated less affinity and depressant effects on myocardial and central nervous vital centres and a superior pharmacokinetic profile [11]. Clinically, levobupivacaine is well tolerated in a variety of regional anaesthesia techniques both after bolus administration and continuous postoperative infusion.

The incidence of adverse events with levobupivacaine was similar to that after bupivacaine in comparative trials. These include hypotension, nausea, postoperative pain, fever, vomiting, pruritus, back pain, headache, constipation, dizziness, and foetal distress [11]. The early clinical presentation of toxicity after levobupivacaine appears to consist of central nervous symptoms (disorientation, drowsiness, slurred speech), which may culminate with tonic-clonic seizures in some cases. Reports of toxicity with levobupivacaine are scarce; occasional toxic symptoms are usually reversible with minimal treatment with no fatal outcome [11].

Surgical sensory block of similar characteristics and recovery over equal dose ranges of levobupivacaine and bupivacaine has been confirmed in surgical patients [11]. The onset of motor block is slower with levobupivacaine, and its quality follows the rank of order bupivacaine > levobupivacaine > ropivacaine [11]. The regression of motor block was significantly more rapid after levobupivacaine and ropivacaine than bupivacaine; this may be advantageous for early ambulation after day-case surgery.

The effective dose of epidural levobupivacaine for continuous postoperative analgesia approaches 15 mg/hour [11]. The addition of adjunctive agents (adrenaline, opioids, or clonidine) to levobupivacaine in epidural anaesthesia and analgesia may increase the duration and quality of analgesia and further decrease the risk of toxicity. Traditionally, the dose of levobupivacaine used for spinal anaesthesia is 15 mg [11]. Smaller doses (5–10 mg) have been used in ambulatory surgery and allow a more rapid recovery and subsequent discharge home.

Current evidence suggests a potency hierarchy of bupivacaine > levobupivacaine > ropivacaine for epidurals in labour [11]. Using an epidural bolus of 10 mL levobupivacaine 0.2%–0.25% followed by epidural infusions or top-ups of low concentrations levobupivacaine (0.1%–0.125%) provides the same good-quality labour analgesia as bupivacaine, but possibly with less motor block [11]. A combined spinal-epidural technique with intrathecal levobupivacaine 1.2–2.5 mg combined with a small dose of opioid (e.g., fentanyl 12.5–25 *μ*g) provides excellent prolonged sensory block with minimum motor blockade [11].

In brachial plexus nerve blocks, a sensory and motor block of similar onset (6–10 min) and duration (14–16 hours) follows the administration of an equal dose of levobupivacaine 0.5% or bupivacaine 0.5% [11]. Continuing the administration of levobupivacaine via a peripheral nerve block continuous catheter is associated with excellent postoperative analgesia as demonstrated by a significant decline in the postoperative systemic opioids requirements.

### 4.2. Ropivacaine

**Figure 5:**
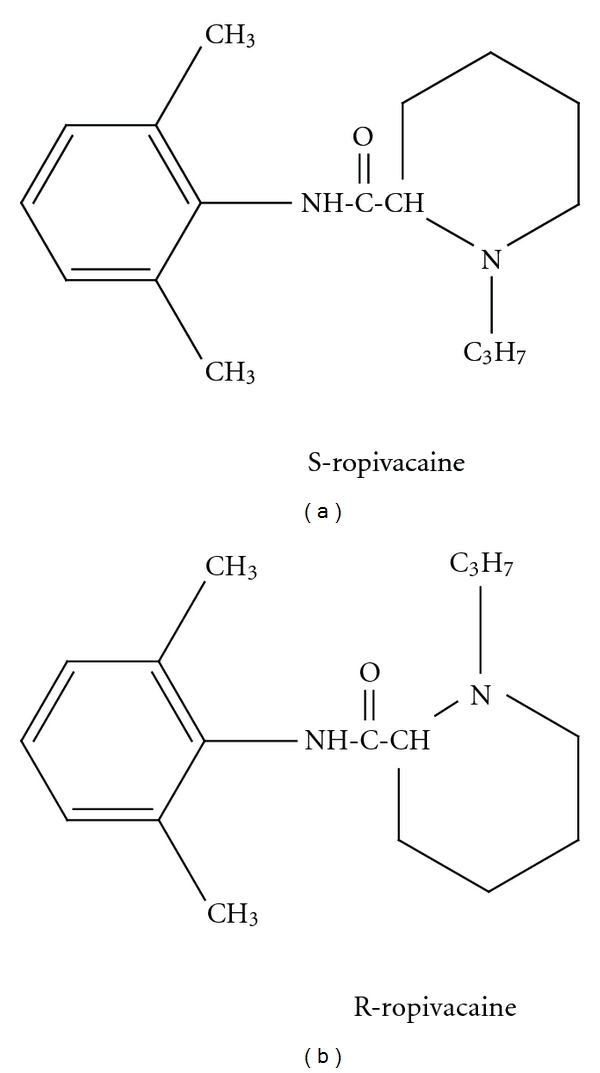
Structure of S and R enantiomers of ropivacaine (marketed as the S enantiomer).

Ropivacaine is a long-acting, enantiomerically pure (S-enantiomer) amide local anaesthetic regional anaesthetic with an efficacy broadly similar to that of bupivacaine (Figure 5). However, it may be a preferred option because of its reduced central nervous system (CNS) and cardiotoxic potential and its lower propensity for motor block [12]. It has a high pKa and low lipid solubility that block nerve fibres involved in pain transmission (A delta and C fibres) to a greater degree than those controlling motor function (A beta fibres). The drug is less cardiotoxic than equal concentrations of racemic bupivacaine but more so than lignocaine; it has a significantly higher threshold for CNS toxicity than racemic bupivacaine. Extensive clinical data have shown that epidural ropivacaine 0.2% is effective for the initiation and maintenance of labour analgesia, and provides pain relief after abdominal or orthopaedic surgery especially when given in conjunction with opioids [12]. Ropivacaine had an adverse event profile similar to that of bupivacaine in clinical trials. Comparative data suggest that higher concentrations of ropivacaine (0.75%) may be needed to provide the same sensory and motor blockade as bupivacaine 0.5% [12]. Brachial plexus anaesthesia was broadly similar to that achieved with equivalent volumes of bupivacaine 0.5%, although the time to onset of sensory block tended to be faster and the duration of motor block shorter with ropivacaine [12].

### 4.3. Articaine

Articaine is a relatively new local anaesthetic used now in dentistry in many countries. It is an amide type local anaesthetic, and, instead of benzene ring, it contains a thiophene ring that increases its lipid solubility. Unlike other local anaesthetics, articaine is exceptional in that it contains an additional ester group that is rapidly metabolised by plasma esterase to articainic acid [13]. As a result, its half-life, about 20 minutes, is also very short compared to other local anaesthetics. Thus, it can rapidly be cleared from the systemic circulation through kidney, minimising adverse effects [13]. Advantages are low lipid solubility, high plasma protein binding rate, fast metabolism, fast elimination half time, and low blood levels [14]. Articaine seems to be the local anaesthetic of first choice in tissues with suppurative inflammation, for adults, children (over 4 years old), elderly, pregnant women, breastfeeding women, and patients suffering from hepatic disorders and renal function impairment [14]. Articaine solutions must not be used in persons who are allergic or hypersensitive to sulphite, due to the content of sodium metabisulphite as the vasoconstrictor's antioxidant in it.

### 4.4. Newer Structurally Different Local Anaesthetics

Another group of potent LAs includes the basic esters of phenylcarbamic acid [15]. Basic esters of alkoxy-substituted phenylcarbamic acid have shown high LA potency, while maintaining a relatively safe toxicity profile [15]. The most potent phenylcarbamic anaesthetics exceed the potency of the most common clinically used local anaesthetics by 100–300 times [15]. Their potency uniquely increases with the decreasing pH of the external medium. This is of importance when using LAs in inflamed tissues, where the action of common LAs is often problematic. Further study of their action is required.

## 5. Mechanisms of Action of Local Anaesthetics

Local anaesthetics directly block transmission of pain from nociceptive afferents. Local anaesthetic agents are applied directly, and their efficacy results from action on the nerve where the inward Na^+^ current is blocked at the sodium ionophore during depolarisation. LAs not only block Na^+^ channels but Ca^2+^ and K^+^ channels [16–18], transient receptor potential vanniloid-1 receptors [19], and other ligand-gated receptors as well. Local anaesthetics also disrupt the coupling between certain G proteins and their associated receptors [20]. Through this action, LAs exert potent anti-inflammatory effects, particularly on neutrophil priming reactions [21]. Local anaesthetics inhibit local inflammatory response to injury that can sensitise nociceptive receptors and contribute to pain and hyperalgesia. Studies have observed that local anaesthetics reduce the release of inflammatory mediators from neutrophils, reduce neutrophil adhesion to the endothelium, reduce formation of free oxygen radicals, and decrease oedema formation [22]. There are, in addition, a variety of other antithrombotic and neuroprotective actions of intravenous LAs [20] that are independent of Na^+^ channel blockade but may account for many of the improvements in pain after surgery [16, 22]. Local anaesthetics can alleviate some types of neuropathic pain, and part of this effect may be related to sensitisation of the antinociceptive pain pathways that occur in the neuropathic pain state; spinal glial cells have been shown to play some part in this as well [23].

Lignocaine seems to have some modulatory effect on the NMDA receptor [24]. Intravenous application of lignocaine in a rat model of acute and neuropathic pain demonstrated antinociception in both pain models [25]. Several studies have previously shown that lignocaine at antiarrhythmic doses or lower doses demonstrates neuroprotective effects [24]. A randomised, double blinded, placebo controlled study of neuroprotection with lignocaine in cardiac surgery showed a potential protective effect of lower lignocaine doses in nondiabetic patients. LAs have long been known to inhibit the growth of different species in vitro [24]. The antibacterial activity of various LAs and additives used in epidural infusions has been tested [26]. Bupivacaine was shown to have the most efficient activity against microorganisms [26]. LAs have been used to enhance bowel function recovery after surgery or trauma. Twenty-two patients scheduled for elective bowel surgery randomised into two groups were given intravenous lignocaine or placebo to assess differences in surgical pain, length of postsurgical ileus, and hospital stay [27]. The lignocaine group showed less pain after 24 hours, a faster return of bowel movements, and an earlier discharge from hospital. LAs stimulate the activity of natural killer cells during the perioperative period [24]. Perioperative lignocaine has been found to improve immediate postoperative pain management and reduce surgery-induced immune alterations [28]. The long-term effect of anaesthesia/analgesia provided by LAs on cancer recurrence needs further investigation.

## 6. Local Anaesthetic Toxicity

Toxicity primarily involves the central nervous system followed by the cardiovascular system. More potent agents (bupivacaine, levobupivacaine, ropivacaine) produce cardiotoxic effects at lower blood concentrations and doses than less potent LA agents (lignocaine) [29]. The (+)-(*R*)-enantiomers bind with greater affinity to cardiac Na^+^ channels than the (−)-(*S*)-enantiomers do. LA agents cause marked but reversible lesions to skeletal muscle tissue [3]. Myotoxicity seems to be explained by mitochondrial bioenergetics alteration. In the animal model, this toxic effect was significantly more severe in young rats [30]. The nitric oxide pathway is involved in the development of tachyphylaxis [31]. In addition, there is a growing amount of evidence that intra-articular administration of bupivacaine is chondrotoxic especially at a higher concentration and with prolonged exposure [24].

A lipid emulsion infusion alongside cardiopulmonary resuscitation appears to be an effective treatment for cardiac toxicity induced by lipophilic medications [32]. The practice advisory on LA systemic toxicity of the American Society of Regional Anesthesia and Pain Medicine suggests that 20% lipid emulsion initially be administered as a bolus of 1.5 mL/kg over a minute [33]. Following completion of the bolus, a continuous infusion of 0.25 mL/kg/min should be started [33, 34]. If the patient does not respond to the initial bolus, one to two additional boluses may be administered. The rate of the infusion may be increased to 0.5 mL/kg if there is persistent hypotension. The infusion should be continued until 10 minutes after the patient regains haemodynamic stability [33, 34]. Given the difficulties of performing clinical trials, further laboratory investigation and clinical correlation are needed to better define its role in resuscitation. Another potential treatment is the use of pegylated anionic liposomes to reduce free drug concentration of LA [35]. In summary, LA cardiotoxicity primarily arises from a blockade of sodium channels. As for treatment, in addition to ventilation, oxygenation, and chest compressions, lipid emulsion therapy should be a primary element in the treatment [36].

### 6.1. Prevention of Local Anaesthetic Toxicity

There is no single measure that can prevent LA toxicity in clinical practice. The lowest effective dose of local anaesthetic should be used (dose = product of volume × concentration). LAs should be injected incrementally through a catheter [33]. The needle or catheter should be carefully aspirated before each injection with close observation when injecting LA. More dilute LAs should be used. The use of ultrasound guidance may reduce the frequency of intravascular injection. Intravascular injection of adrenaline 10–15 microgram/mL in adults produces a >10 beat heart rate increase or a >15-mm Hg systolic blood pressure increase in the absence of beta-blockade, active labour, advanced age, or general/neuraxial anaesthesia [33]. Intravascular injection of adrenaline 0.5 microgram/mL in children produces a >15-mm Hg increase in systolic blood pressure [33]. All these measures have improved morbidity and mortality following LA use.

## 7. Adjuvants

Adrenaline induces vasoconstriction, reducing local anaesthetic clearance from the site of action, thus prolonging the duration of action. Solutions such as 1 : 200000 or 1 : 400000 are commonly used [37].

The addition of bicarbonate raises the pH of LA solution thereby increasing the proportion of unionised LA available to cross the neuronal phospholipid membrane, increasing speed of onset. The recommended dose is 1 mL of 8.4% of sodium bicarbonate per 10 mL of LA. The stability of LAs with added bicarbonate is not well studied; such mixtures cannot be recommended for continuous perineural infusions [37].

Clonidine is an alpha-2 receptor agonist whose effect may be mediated by inhibiting action potentials. Its effect is dose dependent, increasing the duration of anaesthesia and analgesia when used with intermediate acting LAs [37].

Corticosteroids have been shown to specifically inhibit C-fibre transmission [38]. Dexamethasone prolongs peripheral nerve and plexus blocks [5]. It prolongs analgesia from interscalene blocks using ropivacaine or bupivacaine [39], and prolongs the duration of analgesia after supraclavicular brachial plexus blockade using mepivacaine [40].

## 8. Topical Local Anaesthetics

Topical delivery systems for LA are characteristically composed by a diversity of formulations (viscosity inducing agents, preservatives, permeation enhancers, emollients,) and presentations such as semisolid (gel, creams, ointments), liquid (emulsions, dispersions), and solid (patches) pharmaceutical forms [41]. The proposed formulations aim to reduce the LA concentration used, increase its permeability and absorption, keep the LA at the target site for longer and decrease the clearance, and limit local and systemic toxicity [41].

### 8.1. Bioadhesive Plaster and Gels

Different topical local anaesthetics have varying effects on skin blood flow and vascular reactivity. The vasoactive properties of 70 mg lignocaine/70 mg tetracaine medicated plaster (Rapydan), a new topical local anaesthetic, were compared with those of tetracaine base (4.0% w/w or Ametop ) and 2.5% lignocaine/2.5% prilocaine (EMLA ) creams in 20 healthy volunteers [42]. The tetracaine base produced a greater degree of vasodilatation than that seen after the application of a lignocaine/tetracaine medicated plaster [42]. The eutectic patch will be discussed further on.

In the laboratory, the anaesthetic action of the formulated mepivacaine gel containing enhancer and vasoconstrictor was evaluated with the tail-flick analgesimeter [43]. Among the enhancers used, polyoxyethylene 2-oleyl ether showed the greatest enhancement of permeation. The vasoconstrictor tetrahydrozoline showed prolonged and increased local anaesthetic action compared to the control used [43]. Mepivacaine gel is not available commercially for use in humans. Mepivacaine's spinal use was largely abandoned in the late 1990s due to a relatively high incidence of transient neurological symptoms with concentrated mepivacaine solutions [44].

### 8.2. Controlled-Release Local Anaesthetic Matrix

An absorbable, controlled-release lignocaine matrix delivery system has been developed; it is a suspension of a water-insoluble particulate and a hydrophobic carrier containing 16% lignocaine (w/w) (Xybrex ) [45]. It can block the rat sciatic nerve for 1-2 days and suppress postincisional pain [16].

## 9. Encapsulation Matrices

A rapidly growing research topic is the use of vesicular carriers such as liposomes, niosomes, ethosomes (soft lipid vesicles), and elastic and deformable vesicles to provide an efficient dermal delivery system [46]. Encapsulation of local anaesthetic agents allows large doses to be released slowly and provides analgesia over a prolonged period without toxicity [47]. Encapsulating agents include liposomes [48], lipospheres [49], cyclodextrins [50], and microparticles [51]. Following injection of a depot of the formulation, much of the LA agent is bound or carried inside another agent and is not immediately available. The duration of the analgesia depends on the release rate of the LA agent from the carrier agent. Several properties such as hydrophobicity and internal membrane pH affect encapsulated drug release rates [3]. Some synergistic agents such as dexamethasone and clonidine encapsulated with the main effective agent have been formulated; these increased the anaesthesia time for several days [3, 52].

### 9.1. Liposomes

**Figure 6:**
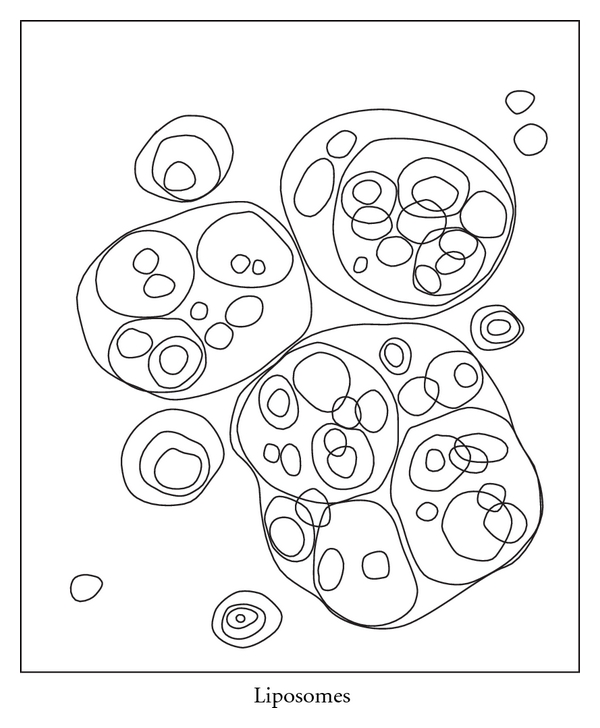
Liposomes.

Liposomes act as reservoirs for drugs. Lipid vesicles are sealed sacs containing a lipid bilayer, usually phospholipids (Figure 6). There are three types of liposomes, namely, multilamellar vesicles, small unilamellar vesicles, and large unilamellar vesicles [3]. Lipid-soluble drugs can be carried in the bilayer itself; liposomes may contain one or more bilayers. Alternatively, aqueous drugs can be carried inside the aqueous compartment contained inside the bilayer [53].

**Figure 7:**
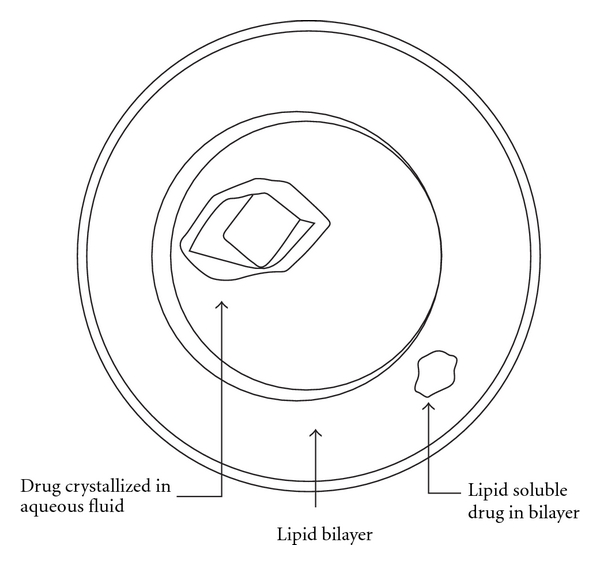
Liposome for drug delivery.

Liposomes supply both a lipophilic region and an aqueous “milieu interior” in one system making them suitable for hydrophobic, aliphatic, and hydrophilic drugs (Figure 7) [54]. They are biocompatible due to their biodegradability and low toxicity. Liposomes help to reduce exposure of sensitive tissues to toxic drugs. They can be administered by a variety of routes (topical, intramuscular, subcutaneous, pulmonary, nasal, oral, and intravenous) [54]. Their route of administration and their lipid composition size can manipulate their pharmacokinetics and in vivo distribution. Following a single injection at the time of surgery, they remain in subcutaneous tissues (around the surgical incision) around a neural plexus or in the epidural space for a much longer period of time compared to the free drug [54].

Liposomes suffer lack of reliability and reproducibility during manufacture owing to oxidation and hydrolysis, which results in leaking of the encapsulated drug [3, 55]. Liposome metabolite compounds have been found to be neurotoxic. The mechanism for this may be lethicin fatty acid oxidation. Also, uncontrolled leakage of drug may occur following breakdown of the liposomes. Ideally, desirable attributes should include prolonged analgesic action; no neurotoxicity; sterility; physical and chemical stability giving a long shelf life; absence of unwanted side products (e.g., residues of organic solvents in bilayer); a reproducible production process [54].

Liposomal formulations of various anaesthetics allow an increase in clinical efficacy in comparison with the plain drugs [56]. Recently, classical liposomes have evolved to “highly deformable” liposomes, endowed with enhanced skin penetration ability and drug skin delivery [57]. “Highly deformable” liposomes consist of phospholipids and an edge activator that is often a single chain surfactant which destabilises the liposomal lipid bilayers, increasing their elasticity and flexibility [58]. Several studies have demonstrated the penetration across the skin of liposomal vesicles to be directly related with their deformability. The high adaptability of such elastic vesicles enable them to squeeze between the cells of the stratum corneum to penetrate intact to the deep layers of the skin; this gives an effect comparable to that of a subcutaneous injection [59].

Limitations of encapsulated local anaesthetic agents include neurotoxicity, myotoxicity, tachyphylaxis, motor block, and viscosity [3]. Many formulations including polymer and liposome carriers have facilitated prolonged local anaesthetic action for several days, although few clinical studies have been performed. Many routes of drug administration have been described, including central neuraxial administration and peripheral nerve administration [3].

Grant et al. [60] described safe and prolonged analgesia for 48 hours following local anaesthetic infiltration of 2% liposomal lignocaine. Boogaerts et al. [61] demonstrated a twofold increase in the duration of the analgesia following epidural administration of liposomal bupivacaine in patients following abdominal surgery. For intraoral topical anaesthesia, liposome-encapsulated 2% ropivacaine gel was as effective as 20% benzocaine gel in reducing pain during needle insertion and inducing soft-tissue anaesthesia; neither, however, was able to induce pulpal anaesthesia [62]. In a recent randomised single-blinded, placebo-controlled (2.5% lignocaine/2.5% prilocaine) cross-over study, liposomal-encapsulated ropivacaine formulations (1%, 2%) did not reduce the pain of insertion of a needle into the palatal mucosa [63]. Another blinded cross-over study in volunteers undergoing intraoral injections at four different sessions evaluated the injection discomfort comparing 2% and 3% liposome-encapsulated mepivacaine with 2% mepivacaine with 1 : 100,000 adrenaline and 3% mepivacaine [64]. The encapsulation of mepivacaine was found to increase the duration of anaesthesia and reduce the injection discomfort caused by these vasoconstrictor-associated formulations [64].

The entrapment of hydrophobic drugs in the aqueous core of liposomes as soluble inclusion complexes with cyclodextrins has been proposed to avoid the use of organic solvents, giving rise to drug-in cyclodextrin-in liposome systems [65]. The main types of cyclodextrin are *α*-, *β*-, and *γ*-cyclodextrins, comprising six to eight sugar units in the ring. This combined approach that simultaneously exploits the cyclodextrin solubilising power towards the drugs and the liposome carrier function through the skin has recently been demonstrated by using both classic [66] and deformable liposomes [67]. The use of this double-loading technique by preparing liposomes loaded with the plain drug in the lipophilic phase and its cyclodextrin complex in the aqueous phase of the vesicles gives rise to a fast onset action and a prolonged effect [68]. The composition vesicles containing the cationic surfactant allowed a significant (*P* < 0.05) improvement of the drug anaesthetic effect in terms of intensity and duration of action [68].

### 9.2. Microemulsions

Microemulsions have penetration-enhancing properties. Local anaesthetics encapsulated in microemulsions result in fast transdermal penetration and effect [39, 69]. Microemulsions contain a large amount of surfactants and cosurfactants that have the potential to cause haemolysis or histopathological changes [69].

### 9.3. Poly(lactic-co-glycolic acid) Microspheres and Nanospheres

The most commonly used micro- and nanoscale vehicles for drug encapsulation and delivery are microspheres and nanospheres. They are usually prepared from biodegradable synthetic hydrophobic materials such as homo- or copolymers of polylactic and polyglycolic acids [70].

**Figure 8:**
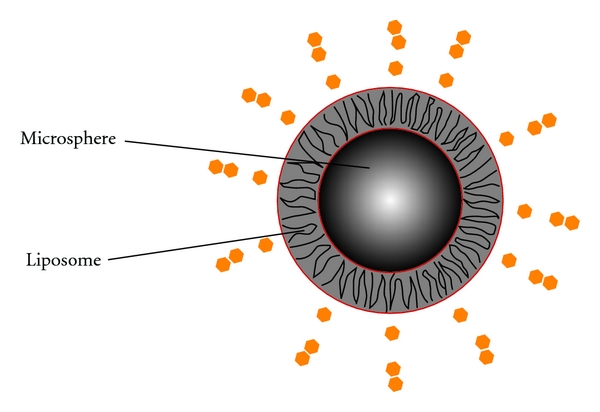
Microspheres (or nanospheres) within liposome.

Microparticles are perfect for drug delivery as they remain at the depot site for long periods allowing slow prolonged release of the encapsulated drug [3]. Particle size and the thickness of thin, free films are factors in drug release. The lipospheres are stable structures consisting of a solid hydrophobic fat core such as triglycerides or fatty acid derivatives, estabilised by a monolayer of phospholipids (Figure 8) [3].

More recently, a nanoliposphere has been developed that does not gelify and is suitable for injection [3, 71]. When poly(dL-lactic acid) microspheres were embedded into poloxamer 407-based hydrogel, this microsphere-gel system containing lignocaine was easy to inject. In addition, it proved degradable [3]. Clinically, in human intercostal blockade studies, dexamethasone added to microcapsules containing bupivacaine showed longer duration of anaesthesia affect compared with microcapsules without dexamethasone [3].

### 9.4. Hyaluronic Acid-Based Hydrogels

Hyaluronic acid is a nonimmunogenic naturally occurring mucopolysaccharide used as a viscous carrier solution to prolong LA action [3]. However, addition of cross-linked hyaluronic acid doubled the length of action of bupivacaine compared with the noncross-linked hyaluronic acid. It is in an easily injectable liquid form.

### 9.5. Calcium Phosphate Apatite Loaded with Bupivacaine

Synthetic calcium-deficient apatites are structurally similar to biological apatites; they are chemical precursors of biphasic calcium phosphates. Biphasic calcium phosphates are mixtures of hydroxyapatite and beta-tricalcium phosphate and are widely used as bone substitutes in human surgery [72]. In Wistar male rats, bupivacaine has been loaded on to calcium-deficient apatites using isostatic compaction [72]. This was able to release local anaesthetic in a manner that prevented or limited postoperative pain following bone surgery [72].

### 9.6. Controlled-Release Local Anaesthetic Matrix

As previously mentioned, the absorbable, controlled-release, local anaesthetic delivery system containing 16% (w/w) lignocaine (Xybrex) is capable of providing up to several days of reversible rat sciatic nerve block in a dose- (mass-) dependent fashion [73]. Two sets of lignocaine-containing drug delivery matrices (OSB-L and OST-R) have been used in subfascial sciatic nerve blocks in rats as well. The OSB-L formulations consisted of four different concentrations of lignocaine ranging from 1.875% to 15% (w/w). These are denoted as OSB-1.875L, OSB-3.75L, OSB-7.5L, and OSB- 15L [73]. The OST-R formulations consisted of four different concentrations of the drug release rate modifier, ranging from 5% to 20% (w/w) in 5% increments, with lignocaine concentration kept constant at 16% (w/w). These formulations are denoted OST-5R, OST-10R, OST-15R, and OST-20R [73]. All OSB-L formulations produced complete and reversible, dose-dependent blockade of nociceptive and motor functions [73]. Blockade by OST-R formulations varied with the concentration of the release rate modifier. All formulations gave complete, reversible blocks of both functions; importantly, their durations did not change monotonically with increasing concentrations of the release rate modifier [73]. Implants of slow-release lignocaine formulations are most effective against postincisional pain when placed at the ipsilateral nerve innervating the area of incision [74]. No human studies of its use have been published as yet.

### 9.7. Injectable Liquid Polymers

There are three types of polymers for encapsulation, namely, nondegradable polymers, synthetic polymers, natural biodegradables (that degrade to nontoxic products that are completely eliminated from the body), and drug-conjugated polymers (where a drug is attached to water-soluble polymer by a cleavable bond) [3]. The drug-polymer conjugate can be directly targeted to the site of specific action. The use of a 15% bupivacaine lactic acid-*co*-castor oil copolymer prolonged the in vivo effect to 96-hour sensory block [75].

Injectable polymers are simple and reliable to prepare; simple mixing combines the local anaesthetic. The main disadvantage is the prolonged time these polymer carriers dwell in the injection site, far beyond the time of the effect of the local anaesthetic agent [3]. The safety and tissue compatibility of biodegradable pasty polymers have been tested and found to be safe with no systemic tissue damage or polymer-related lesions [3, 76].

### 9.8. Films

One of the key areas of intense research is therefore to achieve an optimal and desirable controlled and sustained drug release from the use of biodegradable films. The buccal route has some unique compelling benefits making it worth trying; such benefits include avoiding first pass effect, easy accessibility, and better patient compliance. Adhesion of buccal adhesive drug delivery devices to mucosal membranes leads to an increased drug concentration gradient at the absorption site and therefore improved bioavailability of systemically delivered drugs [77]. Various bioadhesive mucosal dosage forms have been developed as films. An ideal buccal film should be flexible, elastic, and soft, with accepted size and thickness, yet adequately strong to withstand breakage due to stress from mouth activities. It must also possess good bioadhesive strength so that it can be retained in the mouth for a desired duration [78]. It should be nonirritant, not cause teeth discoloration, resistant to metabolism, and be capable of releasing a drug at appropriate rate. Buccal films were developed using carbopol 971P as a mucoadhesive polymer and glycerol as a plasticizer. In testing, a buccal mucoadhesive film using lignocaine and its hydrochloride salt as a model drug found that drug concentration affected the mucoadhesive properties of the films [79].

A slow-release lignocaine sheet has been produced and has been used for sciatic nerve block in the rat model of postoperative pain. Single treatment of this controlled-release lignocaine inhibited hyperalgesia and c-fos expression in the spinal cord dorsal horn for one week without inducing inflammation of the sciatic nerve [80]. Bioadhesive films containing benzocaine have been tested in the rat model for benzocaine local delivery [81]. Tail-flick tests have shown the duration of benzocaine-induced analgesia to be significantly prolonged with the films compared to commercial creams [81].

## 10. Conclusion

Local anaesthetics are widely used to manage acute, chronic, and cancer pain, for anaesthesia, and for diagnostic purposes. Local anaesthetics may have similar chemical structures, but differing pharmacokinetic properties and spectra of pharmacodynamic effects. This influences the selection of agents for use in various clinical situations [82]. New innovations pertaining to LA formulations lead to prolonged action or to novel delivery approaches. Decades after the introduction of local anaesthetics for analgesia/anaesthesia, new properties may still be discovered. New applications of this class of drugs may still be anticipated. The use of regional anaesthesia may affect cancer recurrence rates following surgical resection of tumours via immunomodulation [79]. The preservation of the body's immune processes by local anaesthetics needs to be further studied.The development of new effective delivery systems should suitably modulate the release rate of these drugs, extend their anaesthetic effect, and enhance their localisation; this should reduce problems of systemic toxicity. Part 2 of this paper will deal with new techniques for the delivery of topical and injectable local anaesthetics.
